# Heme Oxygenase-1 Overexpression Promotes Uveal Melanoma Progression and Is Associated with Poor Clinical Outcomes

**DOI:** 10.3390/antiox11101997

**Published:** 2022-10-08

**Authors:** Lucia Longhitano, Giuseppe Broggi, Sebastiano Giallongo, Maria Failla, Lidia Puzzo, Teresio Avitabile, Daniele Tibullo, Alfio Distefano, Valeria Pittalà, Michele Reibaldi, Guido Nicola Zanghì, Antonio Longo, Andrea Russo, Rosario Caltabiano, Giovanni Li Volti, Nicolò Musso

**Affiliations:** 1 Department of Biomedical and Biotechnological Sciences, University of Catania, 95123 Catania, Italy; 2 Department of Medical, Surgical Sciences and Advanced Technologies “G.F. Ingrassia”, Anatomic Pathology, University of Catania, 95123 Catania, Italy; 3 Department of Ophthalmology, University of Catania, 95123 Catania, Italy; 4 Department of Drug and Health Sciences, University of Catania, 95125 Catania, Italy; 5 Department of Molecular Medicine, Arabian Gulf University, Manama 329, Bahrain; 6 Department of Surgical and Medical Specialties, School of Medicine, University of Catania, 95123 Catania, Italy

**Keywords:** uveal melanoma, heme oxygenase, proliferation, carbon monoxide, cancer

## Abstract

Uveal melanoma (UM) is the most common primary intraocular tumor in adults. To date, the main strategies to counteract its progression consist of focal radiation on the tumor site and ocular enucleation. Furthermore, many UM patients develop liver metastasis within 10 years following diagnosis, eventually resulting in a poorer prognosis for those patients. Dissecting the molecular mechanism involved in UM progression may lead to identify novel prognostic markers with significative clinical applications. The aim of the present study was to evaluate the role of Heme Oxygenase 1 (HO-1) in regulating UM progression. UM cell lines (92.1) were treated with Hemin (CONC e time), a strong inducer of HO-1, and VP13/47, a selective inhibitor of its enzymatic activity. Interestingly, our results showed an enhanced 92.1 cellular proliferation and wound healing ability following an HO-1 increase, overall unveiling the role played by this protein in tumor progression. Similar results were obtained following treatment with two different CO releasing molecules (CORM-3 and CORM-A1). These results were further confirmed in a clinical setting using our UM cohort. Our results demonstrated an increased median HO-1 expression in metastasizing UM when compared to nonmetastasizing patients. Overall, our results showed that HO-1 derived CO plays a major role in UM progression and HO-1 protein expression may serve as a potential prognostic and therapeutical factor in UM patients.

## 1. Introduction

Despite its classification as a rare neoplasm, uveal melanoma (UM) is the most common primary intraocular tumor in adults, with about 4.3 cases per million every year [1]. UM progression is characterized by slow and indolent growth, eventually resulting in liver metastasis for 40–50% of cases within 10 years of diagnosis [1]. Unfortunately, metastatic UM patients have a survival rate of 6 to 12 months [2]. In addition, to date no chemotherapeutic regimen or immunotherapy has been reported to be effective at this stage, thus leading to radiation or surgical tumor eradication. To allow for more efficient therapies, understanding the pathobiological processes leading to UM progression and metastasis is needed. In this context, several lines of evidence suggest that heme oxygenase-1 (HO-1) and its biological products (CO and biliverdin) represent a possible target for cancer therapy and to overcome chemoresistance [3,4,5,6,7]. HO-1 is widely expressed at low levels in several tissues, where it catalyzes the degradation of heme to biliverdin, free ferrous iron, and CO, playing a major role in the cellular response to oxidative stress [8]. Within the tumor context, this protein has been previously described as an oncogenic factor eventually protecting cancer cells by scavenging reactive oxygen species (ROS) and in turn increasing upon chemotherapeutic treatments [9,10,11,12,13,14]. In melanoma cells, HO-1 overexpression increases viability, proliferation, and angiogenetic potential, eventually increasing metastasis formation rate and hampering tumor-bearing mouse survival [15,16]. Although both uveal and cutaneous melanomas arise from melanocytes, the former shows peculiar biological and genetic features [17,18]. For this reason, examining the role of the HO-1 system in this context could unveil a novel prognostic marker for UM progression. 

## 2. Materials and Methods 

### 2.1. Cell Culture and Pharmacological Treatments

Human UM cells (92.1) were purchased from ATCC Company (Milan, Italy). Cells were cultured as described in previous studies [19]. Hemin (Sigma–Aldrich, Milan, Italy) was supplemented, where needed, at 10 μM. CORM-3 and CORM-A1 (Sigma–Aldrich, Milan, Italy) were supplemented at 25 μM. VP13/47, namely 1-{4-[(4-bromobenzyl)oxy]phenyl}-2-(1H-imidazol-1-yl) ethanol, was synthesized as reported in previous studies [18] and was supplemented at a final concentration of 50 μM. 

### 2.2. Western Blot Analysis

A western blot analysis was performed as described in previous studies [20]. Briefly, 30 μg of protein extract was loaded onto a 12% polyacrylamide gel MiniPROTEAN^®^ TGXTM (BIO-RAD, Milan, Italy). The blot was then electro-transferred to a nitro-cellulose membrane TransBlot^®^ TurboTM (BIO-RAD, Milan, Italy) using TransBlot^®^ SE semidry transfer cell (BIO-RAD, Milan, Italy) according to manufacturer’s guidelines. Odyssey blocking buffer (Licor, Milan, Italy) was used as blocking solution. Antirabbit primary antibody against HO-1 (1:1000) was purchased from Enzo Life Science (cat. no. BML-HC3001-0025, Milan, Italy). Mouse primary antibody towards β-actin (1:1000) was obtained from Cell Signaling Technology (Cat. No. 4967S, Milan, Italy). Secondary antimouse IRDye800CW (1:5000) (P/N 926-32210) and antirabbit IRDye800CW (P/N: 926-32211) antibodies (1:5000) were obtained from Licor (Milan, Italy). The blots were analyzed using an Odyssey infrared imaging scanner (Licor, Milan, Italy). Protein levels were quantified by densitometric analysis using ImageJ 1.37v (NIH, Maryland, USA). Data were normalized to β-actin expression. 

### 2.3. Real-Time PCR for Gene Expression Analysis 

Real-time PCR samples were prepared as described in previous studies [20]. Briefly, RNA was extracted using Trizol^®^ reagent (Invitrogen, Carlsbad, CA, USA). cDNA was then synthesized by Applied Biosystem’s (Foster City, CA, USA) reverse transcription reagent, according to manufacturer’s guidelines. Real-time PCR was performed in a step one fast real-time PCR system (Applied Biosystems, Foster City, CA, USA), and SYBR green PCR mastermix (Life Technologies, Monza, Italy) was used as detecting agent. The sequence of primers used for HO-1 and actin are presented in Table 1. 

### 2.4. Clonogenic Assay

Clonogenic assay was performed as described in previous studies [20]. Cells were seeded in 6-well plates at low density (3000 cells/well) and they were grown for 9 days. To evaluate their number, colonies were fixed and stained with crystal violet. Quantification was performed using ImageJ 1.37v (NIH, Bethesda, MD, USA).

### 2.5. Wound Healing Assay

Wound healing assay was performed as reported in previous studies [19]. Briefly, 5·10^4^ cells were seeded in 24-well dishes and cultured until confluence. In each dish, a wound was created using a sterile tip. Wound healing ability was then assayed at 0, 4, 8, 24, 48, and 72 h. Quantification of the uncovered wound area was measured using ImageJ 1.37v (NIH, Bethesda, MD, USA).

### 2.6. Real-Time Cell Proliferation Assay

Real-time proliferation was evaluated using xCELLigence (Roche Applied Science, Mannheim, Germany and ACEA Biosciences, San Diego, CA, USA), as described in previous studies [19]. The optimal seeding number (5000 cells/wells) was determined by cell titration and growth experiments. Reagents were supplemented, when needed, 8h after the seeding, in correspondence of cellular log growth phase.

### 2.7. Immunofluorescence

Immunofluorescence assay was performed as established in previous studies [20]. Briefly, before staining, cells were grown on coverslips (Thermo Fisher Scientific, Waltham, MA, USA). Primary HO-1 antirabbit (1:200) was purchased from Enzo Life Science (Cat. No. BMLHC3001-0025, Milan, Italy). FITC antirabbit (1:200) was purchased from Santa Cruz Biotechnology (Cat. No. sc-2012, Santa Cruz Biotechnology, Santa Cruz CA, USA). Nuclear staining was performed using 4’,6-diamidino-2phenylindole (DAPI) (Santa Cruz Biotechnology, Santa Cruz, CA, USA). Data acquisition was performed using a Zeiss Axio Imager Z1 microscope with an Apotome 2 system (Zeiss, Milan, Italy). As a control, immunostaining specificity was tested omitting primary or secondary antibodies incubation. Immunoreactivity was evaluated considering immunofluorescence signal-to-noise ratio.

### 2.8. Patients’ Cohort

Fifty-one primary UMs, which were surgically enucleated at the Ophthalmologic Clinic of the University of Catania, from October 2009 to October 2019, were retrospectively collected. The corresponding clinical pathological data were retrieved from the original pathological reports. For all the cases, enucleation was the only treatment option, being ineligible for plaque brachytherapy or proton beam radiotherapy. Formalin-fixed and paraffin-embedded tissue samples were obtained from the archive of the surgical pathology unit, Department “G.F. Ingrassia”, University of Catania. The present research complied with the Helsinki Declaration and all experiments were approved by the local Ethics Committee, Comitato Etico Catania 1, University of Catania (ID: 003186-24). The previously reported [21] criteria of exclusion were used for case selection.

Hematoxylin and eosin (H&E)-stained slides were separately observed by three pathologists (G.B., L.P., and R.C.), who were not aware of the clinical data of each patient. A series of 25 mUMs and 26 nonmetastatic UMs were included in the study. Clinical parameters, including the tumor’s largest diameter, anatomic location, and metastatic spread (evaluated by ophthalmoscopy, A- and B-scan ultrasounds, liver echography, and whole body computed tomography) were obtained for each case.

### 2.9. Immunohistochemical Analysis

Immunohistochemical tests were performed as described in previous studies [22,23]. Briefly, deparaffinized and pretreated slides were incubated for 30 min at 37 °C with rabbit monoclonal anti-HO-1 (EP1391Y; working dilution 1:200) and mouse monoclonal anti-p16 (CF500036; working dilution 1:150) antibodies. Human unaffected spleen tissue was used as positive control for HO-1, while immunostaining specificity was assayed omitting antibodies incubation. Intensity of staining (IS) and percentage of immunopositive cells (extent score—ES) were assessed by a 0–3 (mild, moderate, and strong) and a 0–4 (< 5%; 5–30%; 31–50%; 51–75%; >75%) scale, respectively. Immunoreactivity score (IRS) was obtained by multiplying IS and ES values. IRS < 6 (L-IRS) indicated low HO-1, while IRS ≥ 6 (H-IRS) indicated high HO-1.

The rates of high and low levels of HO-1 expression in UM patients were nonparametrically compared by a chi-square test. Agreement among observers was tested by Cohen K. Univariate and multivariate analyses were based on a Cox proportional hazards regression model (time free from metastasis as outcome). Gender, age, melanoma location (choroid or ciliary body), temporal or nasal location, cell type (epithelioid, spindle, or mixed cells), echographic parameters (height and greatest diameter), and expression level (low and high) of HO-1 were all included in this model. Any predictor having a *p* value < 0.15 (cut off) in the univariate analysis was included in the multivariate one. Survival analysis according to HO-1 expression level was performed by Kaplan–Meyer test; survival rates were compared by a log rank (Mantel–Cox) test. *p* values < 0.05 were considered as statistically significant.

### 2.10. Statistical Analysis

Statistical analysis was performed using Prism software (Graphpad Software Inc., San Diego, CA, USA), (Graphpad Prism, data analysis software, RRID: rid_000081). Data are expressed as mean ± SEM. Statistical analysis was carried out using an ANOVA test to compare the means of more than two samples.

## 3. Results

### 3.1. Hemin and CORMs Induce HO-1 in UM Cells

**Figure 1:**
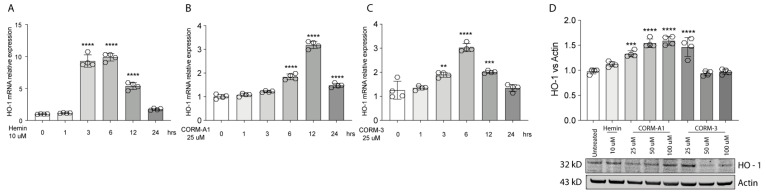
Hemin, CORM-A1, and CORM-3 trigger HO-1 accumulation. (**A**) Hemin (10 μM) increases HO-1 mRNA levels. qPCR analysis showing the increase in HO-1 mRNA levels upon hemin supplementation 3, 6, and 12 h post-treatment. (**B**) CORM-A1 (25 μM) induces HO-1 expression. HO-1 increases its expression 6, 12, and 24 h post-treatment. (**C**) CORM-3 (25 μM) leads to HO-1 overexpression. HO-1 expression 3, 6, and 12 h post-treatment increases post CORM-3 treatment. (**D**) Hemin, CORM-A1, and CORM-3 increase HO-1 protein levels. HO-1 accumulates when 92.1 are treated with different concentrations of Hemin, CORM-A1, and CORM-3. Each result represents the average of four different replicas (mean ± SD). *p* values < 0.05 were statistically significant (** *p* < 0.01; *** *p* < 0.001; **** *p* < 0.0001 vs. 0 h and untreated).

HO-1 is responsible for heme degradation to biliverdin, free ferrous iron, and carbon monoxide and in vitro its activity can be modulated by hemin [3], carrying the porphyrin ring, or by supplementation of CO, in turn mediated by carbon monoxide releasing molecule-3 (CORM-3) or carbon monoxide releasing molecule-A1 (CORM-A1) with different kinetics [24,25]. Consistently, Hemin (10 μM) treatment increases HO-1 expression in 92.1 UM cells. Interestingly, the expression peaks 3 h following treatment, eventually decreasing after 6 h (Figure 1A). Similarly, supplementation of CORM-A1 (25 μM) boosts HO-1 levels, with the highest expression reached after 12 h (Figure 1B). CORM-3 (25 μM), on the other hand, releases CO with a faster kinetic compared to CORM-A1. For this reason, CORM-3 supplementation enhances HO-1 expression, in turn reaching a peak 6 h after the treatment (Figure 1C). Conversely, HO-1 protein levels increase 24 h post-treatment with Hemin (Figure 1C). Mirroring this result, scaling-up CORM-A1 (Figure 1C) and CORM-3 (Figure 1C) concentrations increase accumulation of HO-1 protein levels. Overall, these results show an enhancement of HO-1 mRNA and protein levels in 92.1 UM cells following treatment with hemin, CORM-A1, and CORM-3.

### 3.2. HO-1 Promotes In Vitro UM Proliferation

**Figure 2:**
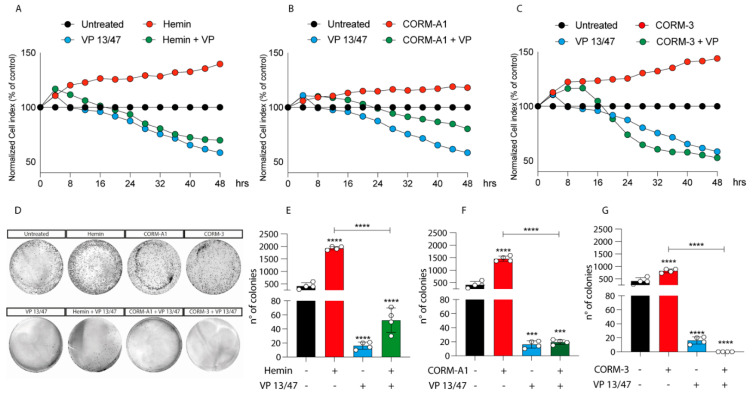
Hemin, CORM-A1, and CORM-3 increase 92.1 proliferation and number of colonies. (**A**) Hemin (10 μM), (**B**) CORM-A1 (25 μM), and (**C**) CORM-3 (25 μM) enhance UM proliferation. xCELLigence assay monitoring real-time 92.1 cell proliferation upon Hemin, CORM-A1, and CORM-3 supplementation, showing an enhanced cellular proliferation already 16, 24, and 20 h post-treatment, respectively Interestingly, VP 13/47 addition (50 μM) disrupts hemin-, CORM-A1-, and CORM-3-, induced proliferation. Each line is expressing the average of four different experiments (mean ± SD). *p* values < 0.05 were statistically significant. (**D**) Clonogenic assay on 92.1 supplemented with Hemin, CORM-A1, and CORM-3. Colony forming ability of 92.1 cells was assayed by Clonogenic assay. (**E**) Hemin (10 μM), (**F**) CORM-A1 (25 μM), and (**G**) CORM-3 (25 μM) enhance 92.1 number of colonies. Clonogenic assay quantification unveiled an increased 92.1 number of colonies following Hemin, CORM-A1, and CORM-3 treatment for 9 days. Of note, VP 13/47 administration (50 μM) disrupted the 92.1 colony forming ability. Each result is representative of four different replicas (mean ± SD). *p* values < 0.05 were statistically significant (*** *p* < 0.001; **** *p* < 0.0001 vs. untreated).

Given the differences in HO-1 dynamics upon Hemin, CORM-A1, and CORM-3 treatment, we decided to investigate the role of HO-1 modulation on UM progression. The proliferation of 92.1 was monitored using xCELLigenece. Considering a 72 h period of time, Hemin (10 μM) increases the proliferation rate of 92.1 UM cells compared to control cells (Figure 2A).

Corroborating the role of HO-1 in promoting UM progression, its selective inhibition, performed by VP13/47 supplementation at a final concentration of 50 μM, resulted in a reduction of cell proliferation (Figure 2A). VP13/47, designed by our research group, is a strongly selective HO-1 inhibitor (HO-1 IC_50_ of 0.95 µM, HO-2 IC_50_ > 100 µM) compared to other inhibitors reported so far [26]. Accordingly, the pharmacological effect of hemin on cell proliferation was abolished by concomitant treatment with VP13/47 (Figure 2A). Therefore, to test whether HO-1 derived CO could be the possible mediator of the observed effects, we also repeated the same set of experiments following the addition of CORM-A1 and CORM-3 (Figure 2B,C). Our results showed that both CORM-A1 (25 μM) (Figure 2B) and CORM-3 (25 μM) (Figure 2C) were able to induce cell proliferation. In agreement with their different CO release kinetic, CORM-3 showed a significantly shorter time of latency compared to CORM-A1 to significantly increase (*p* < 0.01) UM cell proliferation.

The proliferative effect of Hemin and CORMs was further confirmed by clonogenic assay (Figure 2D-G), in turn showing an increased number of colonies following treatment of UM cells with these compounds. Interestingly, VP13/47 treatment impaired Hemin-induced UM clonogenic potential (Figure 2D,E). Mirroring this, VP13/47 supplementation also impaired the number of colonies generated by UM cells upon CORMs treatment (Figure 2F,G).

**Figure 3:**
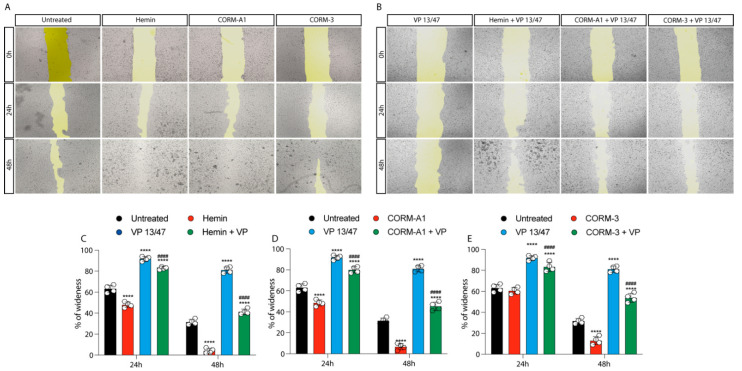
Hemin and CORMs increase 92.1 wound healing ability. (**A**) 92.1 supplemented with Hemin (10 μM), CORM-A1 (25 μM), and CORM-3 (25 μM) increase their wound closure speed. The differences are already visible 24 h post-treatment. (**B**) VP 13/47 (50 μM) impairs Hemin- and CORMs- induced 92,1 wound closures. The opening of the wound is drastically affected by VP 13/47 supplementation, eventually hampering hemin, CORM-A1, and CORM-3-induced closure. Quantification of the percentage of wideness resulting from (**C**) Hemin (10 μM), (**D**) CORM-A1 (25 μM), and (**E**) CORM-3 (25 μM) supplementation. The three molecules, once administered, increase 92.1 wound closure. Their effect is disrupted by VP 13/47 (50 μM) addition. Each result is representative of four different replicas (mean ± SD). *p* values < 0.05 were statistically significant **** *p* < 0.0001 vs. untreated. ^####^ *p* < 0.0001 vs. (**C**) Hemin, (**D**) CORM-A1, and (**E**) CORM-3).

The role of HO-1 in UM proliferation was further confirmed by a wound healing assay, in turn displaying a faster wound closure timing following Hemin treatment compared to control UM cells (Figure 3A–C).

This effect was abolished by VP13/47 (50 μM) treatment (Figure 3A–C). Corroborating these data, CORMs increased 92.1 UM wound closure ability compared to their respective controls (Figure 3A,B,D,E). Notably, CORMs’ difference in CO release kinetics was mirrored by their ability in rescuing VP13/47 decreased proliferation. Interestingly, whereas CORM-A1 did not rescue UM proliferative potential (Figure 3A,B,D), CORM-3 was able to counteract VP13/47 reduced cell proliferation within a time window of 24h (Figure 3A,B,E).

Examined together, our results demonstrated that HO-1 enzymatic activity, in turn mediated by Hemin and CORMs, eventually enhances UM cells proliferation. Interestingly, HO-1 pharmacological inhibition, mediated by VP13/47, disrupt Hemin and CORM-A1 mediated phenotypes, demonstrating a slower dynamic upon CORM-3-induced proliferation.

### 3.3. CORMs Enhance UM Mitochondrial Fitness

**Figure 4:**
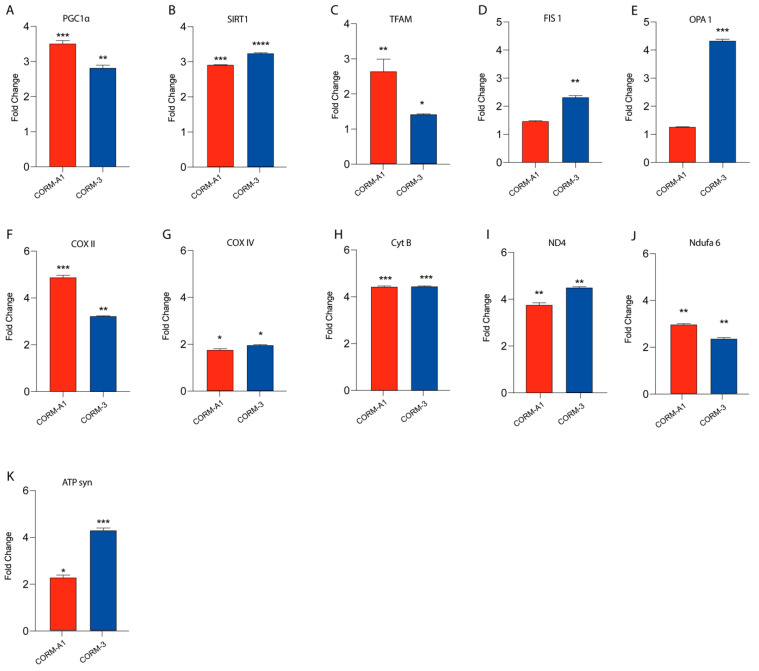
CORMs (24 μM for 24 h) supplementation increases 92.1 mitochondrial fitness. (**A**–**K**) qPCR analysis unveils an increased expression of genes involved in UM mitochondrial fitness. (**A**) PGC1α, (**B**) SIRT1, (**C**) TFAM, (**D**) FIS1, (**E**) OPA1, (**F**) COX II, (**G**) COX IV, (**H**) CytB (**I**) ND4, (**L**) Ndufa6, and (**M**) ATP syn show an increase in expression upon CORM-A1 and CORM-3 supplementation. Each result is representative of four different replicas (mean ± SD). *p* values < 0.05 were statistically significant (* *p*< 0.05; ** *p*<0.01; *** *p* < 0.001; **** *p* < 0.0001 vs. untreated).

Our previous findings linked HO-1 derived CO to UM proliferation. Notably, these results were also mirrored by CORMs supplementation, which have been reported to exacerbate an outstanding role as a ROS scavenger, in turn releasing CO. In this context, we decided to investigate if CORMs supplementation improves mitochondrial fitness in UM cells. Our results, performed by qPCR, showed that the addition of CORM-A1 and CORM-3 enhanced the expression of PGC1α, SIRT1, TFAM, FIS1, OPA1, COXII, COXIV, CYTB, ND4, NDUFA6, and ATP-SYNTHASE compared to control UM cells (Figure 4A–K).

Overall, these data corroborate the hypothesis supporting the role of CORMs as pharmacological treatments enhancing UM progression by boosting their mitochondrial fitness.

### 3.4. HO-1 Expression Is a Prognostic Marker of Mortality and Correlates to Disease Stage

The clinical pathological characteristics of UM cases from our cohort are listed in Table 2 and Table 3. Fifty-one patients (27 males and 24 females) with an age ranging from 19 to 85 years (median age: 69 years) were part of the present study. UMs were exclusively located at the choroid in 39 cases, while a concomitant involvement of choroid and ciliary bodies was present in 12 cases. Only one case exhibited extrascleral invasion at diagnosis. Out of 51 cases, 27 were histologically diagnosed as mixed cell type, while “pure” epithelioid cell and spindle cell morphologies were found in 16 and 8 cases, respectively. Twenty-five patients developed liver metastases. The median follow-up period was 73 months (range: 1–168). Tumors showed the following pT stages: pT1a in one case, pT1b in one case, pT2a in twenty-one cases, pT2b in ten cases, pT3a in eleven cases, pT3b in two cases, pT3d in one case, pT4a in two cases, and pT4b in the remaining two cases. The group of UMs without metastases included 26 patients (Table 2) (16 males and 10 females) with a median age of 67 years (age range: 19–81 years). Among the 25 metastatic patients, 11 were males and 14 females with a median age of 71 years (age range: 48–85). Of the 25 patients with liver metastases, 17 died from the disease during the follow-up time.

When comparing metastasizing and nonmetastasizing UMs (Table 2 and Table 3), no significant difference in median age, melanoma anatomic location (choroid or choroid and ciliary body), melanoma thickness, cell type, extrascleral extension, and pT stage was detected. On the other hand, metastatic disease patients presented a neoplasm characterized by a greater median largest diameter (15.4 mm versus 13.3 mm, *p* = 0.102), and a higher median HO-1 expression (8 versus 2, *p* < 0.001 and shorter median disease-free survival (24 months versus 86 months, *p* < 0.001) (Table 3).

**Figure 5:**
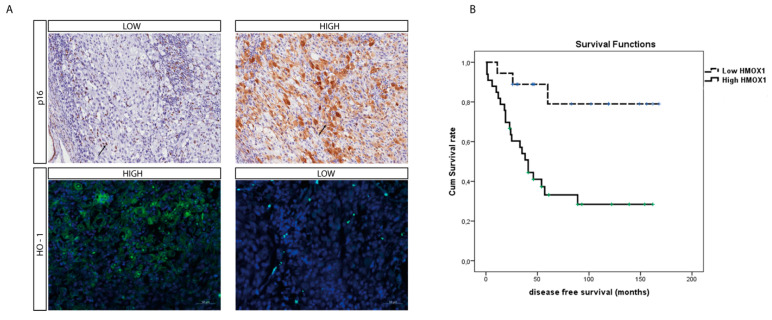
O-1 accumulation correlates with higher-risk UM. (**A**) Immunohistochemical expression of HO-1 anticorrelates with p16 expression in an epithelioid cell-type UM. HO-1 expression increases with the progression of this tumor, as showed by the anticorrelation with the tumor suppressor protein p16. (immunoperoxidase; original magnification 200×). (**B**) Kaplan–Meier survival curves in UM patients. Disease-free survival decreases when HO-1 is accumulating.

To further assess the role of HO-1 as a UM prognostic marker, we evaluated its protein level within the UM cohort using immunohistochemistry. Interestingly, the median HO-1 value was four (range: 0–12); HO-1 IRS was high in 32 cases and low in 19 cases, in turn inversely correlating with the p16 signal (Figure 5A) whose immunohistochemical expression on our UM series is summarized in Table 2 and Table 3

Out of 26 free-of-metastasis patients, 15 (57.7%) exhibited HO-1 L-IRS, while the remaining 11 cases (42.3%) had HO-1 H-IRS (Fisher’s exact test, *p* = 0.003, Table 4). Conversely, high HO-1 immunohistochemical expression was found in 21 out of 25 metastasizing cases (84%), while HO-1 L-IRS was observed in only 4 cases (16%) (Fisher’s exact test, *p* = 0.003, Table 5). Factors related to the presence of metastasis at univariate analysis on a Cox proportional hazards regression model included thickness (*p* = 0.090), largest diameter (*p* = 0.068), epithelioid cell type (*p* = 0.116), pT stage (*p* < 0.001), and HO-1 level (*p* = 0.006). In the multivariate analysis, the pT stage (*p* = 0.017) and HO-1 level (*p* = 0.028) were significant. No correlation was found between histological type and HO-1 expression (Spearman’s rho *p* = 0.632).

Furthermore, the mean survival times free from metastasis (SE, with 95% CI) estimated were respectively: 140.7 (14.3) (CI: 122.6 to 168.8) and 67.5 (11.4) (CI: 45.1 to 89.9), as showed by Kaplan–Meier survival analysis (Figure 5B). The log rank test showed a significant difference (*p* = 0.002) between the two groups.

In a Cox survival analysis, factors related to survival time free from metastasis were HO-1 level (*p* < 0.001), pT stage (0.006) and cell type (*p* = 0.006). Additionally, after adjusting for age, sex, and pT stage, HO-1 level and cell type were related to survival time free from metastasis (respectively *p* < 0.001 and *p* = 0.004).

## 4. Discussion

To date, UM represents the most occurrent primary ocular malignancy. The main strategies to treat UM involve brachytherapy, operated by suturing a radioactive plaque to the sclera to deliver focal radiation to the tumor site, and ocular enucleation [27]. If this is unsuccessful, UM patients develop liver metastasis with a survival rate from 6 to 12 months [2]. For this reason, investigating the mechanisms promoting UM could lead to the identification of novel therapeutic targets and diagnostic markers. In the present study we investigated the role of HO-1 as a potential contributor towards UM progression. Previous reports showed a significative prognostic value in breast cancer, suggesting that increased HO-1 expression is associated to a shorter disease-free survival, a lower pathological complete response, and a worse overall survival [18,28]. To investigate the role of HO-1 as UM prognostic marker we first assessed its enzymatic activity in an in vitro model. For this purpose, we used its inducer Hemin, observing an increased HO-1 protein and mRNA expression as described in previous studies [3]. HO-1 activity leads to the oxidative cleavage of heme groups generating biliverdin, ferrous iron, and CO [5,29,30]. The latter can be administered to cells by CORMs. Here, we supplemented two different substrates, CORM-A1 and CORM-3, eventually releasing CO with different kinetics. Our results showed an enhanced HO-1 mRNA and protein levels following the addition of CORMs, agreeing with another study where an increased neurogenic differentiation as a result of CORMs-mediated HO-1 activation was described [24,25,31]. To further assess the role of HO-1 within the UM context, we performed in vitro assays aiming to investigate UM cell proliferation upon HO-1 activation. Our results described an enhanced cell proliferation orchestrated by Hemin, CORM-A1, and CORM-3, eventually triggering HO-1 activity. Interestingly, treatment with CORMs rescued the proliferative phenotype of UM cells following treatment with the specific inhibitor of HO-1 enzymatic activity thus suggesting that HO-1 derived CO is responsible for the observed effects rather than other byproducts or protein expression.

These results agree with the data described in a different melanoma model, where HO-1 was reported to interact with B-Raf, eventually triggering the B-RAF-ERK1/2 signaling pathway leading to CDK2/cyclin E activation, thus promoting melanoma proliferation [32,33]. Furthermore, the clinical course of xenograft mice post-intracutaneous inoculation of melanoma cells overexpressing HO-1 demonstrated a higher percentage of tumors with increased vascularization and VEGF production. Moreover, they were characterized by a lower grade of inflammatory edemas, leukocyte infiltration with accumulating soluble receptor 1 of TNF-α (sTNF-α R1). As a result, these data showed the inverse correlation standing between HO-1 expression and mice survival [15,34]. Corroborating this data, targeting HO-1 showed significant potential to counteract metastatic melanoma, given the outstanding role of Nrf2 and HO-1 enzymatic activity in melanosphere formation [35]. More broadly, HO-1 has been reported as an outstanding player promoting cellular proliferation in prostate, pancreatic, and hepatoma cancer cells [36,37]. In these models, HO-1 upregulation promotes cancer invasiveness, eventually enhancing the vascular endothelial growth factor (VEGF) axis [38]. Corroborating the role of HO-1 as oncoprotein, in colon cancer cells the glucose-regulated protein 78 (GRP78) enhances migration and invasiveness by inducing vimentin expression, in turn reducing E-cadherin level, and triggering Nrf2/HO-1 signaling [39].

Overall, these results underlined the outstanding role played by HO-1derived CO in promoting UM progression, thus representing a valuable prognostic marker to use in clinical application. Therefore, in order to translate our in vitro results on a single cell line in a clinical setting we further assessed HO-1 expression in tissue obtained from UM patients.

The comparison between metastasizing and nonmetastasizing UMs was characterized by an increased median HO-1 expression and also corroborated by an immunohistochemical analysis, overall supporting the role of HO-1 as an oncoprotein. Interestingly, immunohistochemical analysis inversely correlated p16 and HO-1 accumulation. Our ex vivo data significatively correlated with a previous report published by Luo et al., which eventually designed a ferroptosis-related seven-gene signature of UM based on patients’ outcomes [40,41]; Among them, the authors found that HO-1, along with STEAP3, ITGA6, and AIFM2/FSP1, was an unfavorable gene for UM outcome, which is in agreement with our data [40].

## 5. Conclusions

In conclusion, our work describes the important role played by HO-1 in UM progression and suggests it as a possible biomarker to evaluate UM progression.

## Figures and Tables

**Table 1:** Primer sequences used for qPCR analysis.

Primer	Forward (5’→3’)	Reverse (5’→3’)	Accession Number
HMOX1	AAGACTGCGTTCCTGCTCAA	GGGCAGAATCTTGCACTTTGT	NM_002133.3
PGC1alpha	ATGAAGGGTACTTTTCTGCCCC	GGTCTTCACCAACCAGAGCA	NM_001330751.2
SIRT1	AGGCCACGGATAGGTCCATA	GTGGAGGTATTGTTTCCGGC	NM_012238.5
TFAM	CCGAGGTGGTTTTCATCTGT	AGTCTTCAGCTTTTCCTGCG	NM_003201.3
FIS1	CTGCTCCCCTGAGATTCGTC	AGCCACAGCCCCGTTTTATT	NM_016068.3
OPA1	CTGTGGCCTGGATAGCAGAA	GCGAGGCTGGTAGCCATATT	NM_001354663.2
COX IV	CAGCTCTCGGAAGCGTTGTA	GATAACGAGCGCGGTGAAAC	NM_001318802.2
CyT B	TCTTGCACGAAACGGGATCA	TGATTGGCTTAGTGGGCGAA	MZ475297.1
ND4	ACAAGCTCCATCTGCCTACGACAA	TTATGAGAATGACTGCGCCGGTGA	MZ475297.1
Ndufa 6	GATGCTTTGGCAAGATGGCG	GTCCCGACTGAAAATGGGCT	NM_002490.6
ATP synthase	CCGCCTTCCGCGGTATAATC	ATGTACGCGGGCAATACCAT	NM_001001937.2
Beta Actin	CCTTTGCCGATCCGCCG	AACATGATCTGGGTCATCTTCTCGC	NM_001101.5

**Table 2:** Demographics, tumor parameters, disease-free time, follow-up, and HO-1 expression in primary uveal melanoma without metastasis (n = 26).

Sex	Age (yrs)	Location	Thickness (mm)	Largest Diameter (mm)	Cell Type	Extrascleral Extension	Pathological T Stage	DFS (Months)	Follow-Up (Months)	HO-1				p16			
										IS	ES	IRS (High ≥ 6; Low < 6)		IS	ES	IRS (High ≥ 6; Low < 6)	
f	29	ch	14.20	16.20	mixed	N	pT2a	168	168	1	1	1	L	2	4	8	H
f	30	ch/cb	12.05	9.20	spindle	N	pT2a	162	162	1	1	1	L	3	3	9	H
f	55	ch	9.80	13.90	spindle	N	pT2a	162	162	2	3	6	H	2	2	4	L
m	68	ch	12.80	20.10	mixed	N	pT2b	156	156	2	1	2	L	2	3	6	H
m	64	ch	7.70	11.50	epit	N	pT2a	154	154	2	2	4	H	1	3	3	L
f	36	ch	5.81	12.70	spindle	N	pT2b	149	149	0	0	0	L	2	2	4	L
m	58	ch	13.10	14.30	mixed	N	pT2a	139	139	2	4	8	H	2	2	4	L
m	63	ch	3.30	11.70	spindle	N	pT2a	122	122	2	2	4	H	2	1	2	L
m	74	ch/cb	10.04	16.10	spindle	N	pT2b	119 (†)	119 (†)	0	0	0	L	3	3	9	H
m	62	ch	6.32	10.00	epit	N	pT2b	119	119	2	1	2	L	2	4	8	H
m	73	ch	9.70	11.30	mixed	N	pT1a	102 (†)	102 (†)	1	1	1	L	2	2	4	L
m	80	ch	9.24	17.70	epit	N	pT2a	93 (†)	93 (†)	2	3	6	H	2	2	4	L
f	78	ch	8.07	10.70	mixed	N	pT2a	89	89	2	3	6	H	1	2	2	L
m	81	ch	7.38	9.86	mixed	N	pT2a	83 (†)	83 (†)	2	1	2	L	2	1	2	L
f	57	ch	10.00	11.24	mixed	N	pT2a	61	61	3	3	9	H	3	3	9	H
m	66	ch/cb	13.50	17.50	epit	N	pT3a	54	54	2	2	4	H	2	2	4	L
m	74	ch	8.02	14.61	epit	N	pT3b	47	47	1	1	1	L	3	4	12	H
f	52	ch	4.96	9.92	spindle	N	pT3a	46	46	1	1	1	L	3	2	6	H
m	79	ch	12.28	14.95	mixed	N	pT2a	46	46	2	3	6	H	3	3	9	H
m	67	ch	7.8	11.3	spindle	N	pT2a	45	45	2	1	2	L	2	3	6	H
m	64	ch	9.30	15.17	mixed	N	pT2b	41	41	2	2	4	H	3	3	9	H
m	19	ch	9.77	14.76	mixed	N	pT3b	31	31	2	1	2	L	3	3	9	H
m	73	ch	15.89	18.00	mixed	N	pT2a	30	30	1	1	1	L	2	3	6	H
f	81	ch/cb	8.12	11.37	mixed	N	pT3a	26	26	2	1	2	L	2	4	8	H
f	80	ch	14.61	14.30	epit	N	pT2b	26	26	2	3	6	H	2	2	4	L
f	69	ch	8.73	11.38	mixed	N	pT2a	23	23	2	1	2	L	3	3	9	H

Abbreviations: IS, intensity of staining; ES, extent score; IRS, immunoreactivity score; L, low; H, high; † dead patient.

**Table 3:** Demographics, tumor parameters, disease-free time, follow-up, and HO-1 expression in primary uveal melanoma with metastasis. (n = 25).

Sex	Age (yrs)	Location	Thickness (mm)	Largest Diameter (mm)	Cell Type	Extrascleral Extension	Pathological T Stage	DFS (Months)	Follow-Up (Months)	HO-1				p16			
										IS	ES	IRS (High ≥ 6; Low < 6)		IS	ES	IRS (High ≥ 6; Low < 6)	
m	76	ch	13.7	17.1	mixed	N	pT2a	14	119	2	1	2	L	3	3	9	H
f	50	ch	7.36	15.6	epit	N	pT2a	41	111	2	3	6	H	2	2	4	L
m	48	ch/cb	15.34	14.3	mixed	N	pT4b	41	105 (†)	2	4	8	H	2	2	4	L
f	57	ch/cb	13.6	19	epit	N	pT2b	6	96	2	2	4	H	2	4	8	H
f	51	ch	9.42	19.03	mixed	N	pT3a	25	96	3	3	9	H	1	2	2	L
f	84	ch	11.7	17.4	mixed	N	pT2b	89	91 (†)	2	4	8	H	2	1	2	L
f	74	ch	11.35	18.5	mixed	N	pT4a	19	87	3	3	9	H	2	2	4	L
f	66	ch/cb	8.95	15.4	mixed	N	pT2b	12	84	3	4	12	H	2	2	4	L
m	69	ch	7.21	15.8	mixed	N	pT2a	54	81 (†)	3	3	9	H	3	1	3	L
m	71	ch	11.69	16.63	mixed	N	pT3a	57	81 (†)	2	4	8	H	2	2	4	L
f	54	ch/cb	9.76	11.5	mixed	N	pT2b	46	74 (†)	3	3	9	H	2	3	6	H
f	64	ch/cb	3	6	epit	N	pT1b	60	73 (†)	2	2	4	H	2	2	4	L
m	73	ch	9.6	15	epit	N	pT3a	10	60	2	4	8	H	2	2	4	L
f	74	ch/cb	15.93	19.7	mixed	N	pT2a	35	53 (†)	1	3	3	L	1	2	2	L
m	62	ch	13.68	14.7	mixed	N	pT3a	38	51 (†)	2	4	8	H	3	2	6	H
f	74	ch	5.7	12.1	spindle	N	pT2a	24	50 (†)	2	4	8	H	1	2	2	L
f	85	ch/cb	7.3	12.5	epit	Y	pT3d	26	49 (†)	2	4	8	H	2	2	4	L
m	71	ch	13.14	17.1	epit	N	pT3a	33	46 (†)	2	4	8	H	2	2	4	L
f	76	ch	10.81	12.52	mixed	N	pT3a	23	42	1	1	1	L	3	4	12	H
m	70	ch	9.87	11.16	epit	N	pT3a	19	37 (†)	2	4	8	H	1	3	3	L
f	60	ch	8.25	16.5	epit	N	pT2a	11	25 (†)	2	2	4	H	2	3	6	H
m	81	ch	13.9	12	mixed	N	pT3a	18	19 (†)	2	4	8	H	2	2	4	L
f	48	ch	10.71	12.86	epit	N	pT3a	1	10 (†)	2	1	2	L	3	3	6	H
m	78	ch	16.58	16.59	epit	N	pT4a	2	3 (†)	2	4	8	H	2	4	8	H
m	72	ch/cb	13.3	15.4	mixed	N	pT3b	1	1 (†)	2	4	8	H	2	1	2	L
														3	3	9	H

Abbreviations: IS, intensity of staining; ES, extent score; IRS, immunoreactivity score; † dead patient.

**Table 4:** Median (range) of demographics, tumor parameters, disease-free time, follow-up, and HO-1 expression in primary uveal melanoma with and without systemic metastasis.

	Sex m-f	Age (yrs)	Location	Thickness	Largest diameter	Cell Type	Extrascleral Extension	Pathological T Stage	DFS (months)	Follow-Up (months)	HO-1
All (n = 51)	27–24	69 (19–85)	ch 39 ch/cb 12	9.8 (5.7–14.2)	14.6 (9.2–20.1)	Epith: 16 Spindle: 8 Mixed: 27	No: 50 Yes: 1	pT1a: 1 pT1b: 1 pT2a: 21 pT2b: 10 pT3a: 11 pT3b: 2 pT3d: 1 pT4a: 2 pT4b: 2	41 (1–168)	73 (1–168)	4 (0–12)
Metastasis free (n = 26)	16–10	67 (19–81)	ch 22 ch/cb 4	9.5 (3.3–15.9)	13.3 (9.2–20.1)	Epith: 6 Spindle: 7 Mixed:13	No: 26	pT1a: 1 pT2a: 15 pT2b: 6 pT3a: 2 pT3b: 2	86 (23–168)	86 4 death (23–168)	2 (0–9)
Metastasis (n = 25)	11–14	71 (48–85)	ch 17 ch/cb 8	10.8 (3–16.9)	15.4 (6.0–19.7)	Epith: 10 Spindle: 1 Mixed: 14	No: 24 Yes: 1	pT1b: 1 pT2a: 6 pT2b: 4 pT3a: 9 pT3b: 1 pT3d: 1 pt4a: 2 pT4b: 1	24 (1–63)	60 17 death (1–119)	8 (1–12)
p (metastasis free vs. metastasis)		0.820 *	0.506	0.392 *	0.102 *	0.859 *	0.490	0.064 *	0.001 *	0.0973 *	<0.001*

**Table 5:** Number of uveal melanoma (with and without metastasis) with low and high HO-1 levels.

	Metastasis (n = 25)	Metastasis free (n = 26)
Low	4 (16.0%) *	14 (53.8%)
High	21 (84.0%)	11 (42.3%)

## Data Availability

All of the data is contained within the article.
